# How an Interest in Mindfulness Influences Linguistic Markers in Online Microblogging Discourse

**DOI:** 10.1007/s12671-023-02098-4

**Published:** 2023-03-17

**Authors:** Clara Eugenia Rivera, Rebekah Jane Kaunhoven, Gemma Maria Griffith

**Affiliations:** Centre for Mindfulness Research and Practice, School of Human and Behavioural Sciences, Bangor University, Brigantia Building, Penrallt Road, Bangor, LL57 2AS UK

**Keywords:** Mindfulness, Twitter, Psycholinguistics, LIWC, Language, Linguistic markers, Speech, Behaviour

## Abstract

**Objectives:**

This study aimed to investigate the linguistic markers of an interest in mindfulness. Specifically, it examined whether individuals who follow mindfulness experts on Twitter use different language in their tweets compared to a random sample of Twitter users. This is a first step which may complement commonly used self-report measures of mindfulness with quantifiable behavioural metrics.

**Method:**

A linguistic analysis examined the association between an interest in mindfulness and linguistic markers in 1.87 million Twitter entries across 19,732 users from two groups, (1) a mindfulness interest group (*n* = 10,347) comprising followers of five mindfulness experts and (2) a control group (*n* = 9385) of a random selection of Twitter users. Text analysis software (Linguistic Inquiry and Word Count) was used to analyse linguistic markers associated with the categories and subcategories of mindfulness, affective processes, social orientation, and “being” mode of mind.

**Results:**

Analyses revealed an association between an interest in mindfulness and lexical choice. Specifically, tweets from the mindfulness interest group contained a significantly higher frequency of markers associated with mindfulness, positive emotion, happiness, and social orientation, and a significantly lower frequency of markers associated with negative emotion, past focus, present focus, future focus, family orientation, and friend orientation.

**Conclusions:**

Results from this study suggest that an interest in mindfulness is associated with more frequent use of certain language markers on Twitter. The analysis opens possible pathways towards developing more naturalistic methods of understanding and assessing mindfulness which may complement self-reporting methods.

In his *Tractatus Logico-Philosophicus*, Wittgenstein (1961) famously wrote “the limits of my language mean the limits of my world” (p. 23). As a system of symbols to express thoughts and feelings, language and words reveal important information about us. How we express ourselves reflects who we are, what we feel, how we process information, and what we care about. For example, people experiencing positive emotions use more positive affect words and more exclamation marks (Hancock et al., 2007), whilst those in pain tend to focus their attention on themselves and use more first-person singular pronouns (Rude et al., 2004). Therefore, the study of language and words can help understand the human mind.

Early attempts to link language use and psychological states followed difficult-to-scale approaches that required recording subjects, transcribing speech, and training groups of evaluators to review, count, identify phenomena, and categorise samples (Gleser et al., 1961). However, increased computing power and the growing availability of samples obtained through the internet is revolutionising computational psycholinguistics with advanced systems that are capable of processing and analysing data in unprecedented ways (Church & Mercer, 1993). An increasing body of literature has explored linguistic markers and their connection with consumer behaviour (Puschmann & Powell, 2018), political preferences (Abe, 2018), effective corporate leadership (Scheuerlein & Chládková, 2019), and radical violence (Kaati et al., 2016). In the field of mental health, the study of linguistic markers has enabled the identification of differences between healthy subjects and subjects with mental health issues (Cummins et al., 2015; Ringeval et al., 2017), predictions of mental health disorders (De Choudhury et al., 2013; Stasak et al., 2017), and early diagnosis and monitoring of high-risk populations (Pestian et al., 2017).

The internet has increased the availability of psycholinguistic data sources by several orders of magnitude. Such sources include natural language from social media, online news, readers’ comments, customer reviews of products and services, blogs, forum discussions, and messaging apps, and have allowed research in this field to expand in scale and scope (Kraut et al., 2004). The vast extent of its dataset, with more than 500 million tweets being created every day and around 90% of accounts being publicly accessible (Batrinca & Treleaven, 2015), makes Twitter an excellent data source for research. Whilst Twitter language presents intrinsic characteristics related to brevity (e.g., abbreviations) and register (e.g., casual tone), research has shown that it is quite similar to the language used on other online platforms like news or blogs (Hu et al., 2013). Moreover, from a lexical frequency perspective, words of choice tend not to vary significantly when compared to other data sources (Gimenes & New, 2016) and from a topical and narrative perspective Twitter language shares characteristics with other formats of recounting experience such as diaries (Humphreys et al., 2013). Some research points to Twitter data as providing better word frequency for psycholinguistics over other sources of written text such as news or subtitles (Herdağdelen & Marelli, 2017). Whilst Twitter increased the maximum character count from 140 to 280 in 2017, the language used did not experience many differences and only 5% of tweets went beyond 190 characters in English (Perez, 2018) but also in other languages like Dutch (Boot et al., 2019). This has favoured early explorations of Twitter language in relation to a broad range of psychosocial traits, and one study found it was possible to predict Twitter users’ Big Five personality traits from their Twitter account (Golbeck et al., 2011). Other researchers have examined Twitter use and how it relates to depression and mental health detection (Guntuku et al., 2017), post-traumatic stress disorder (Coppersmith et al., 2014), schizophrenia (Mitchell et al., 2015), and the impact of strict COVID-19 lockdowns in Wuhan and Lombardy (Su et al., 2020). In one study from the USA, the language used on Twitter with certain psychological characteristics (e.g., anger, negative emotion language, disengagement) was associated with heart disease mortality risk, and their counterparts (engagement, positive emotion language) were protective at a county-wide level (Eichstaedt et al., 2015). There is a growing literature base on psychological constructs and behaviour on Twitter, although the field of language markers and mindfulness remains almost unexplored.

Mindfulness is commonly defined as “paying attention in a particular way: on purpose, in the present moment, and nonjudgmentally” (Kabat-Zinn, 1994, p. 4). Mindfulness can be considered a state which is either fostered through mindfulness practice or a disposition which is the natural ability to attend in an open and non-judgemental way to experiences within daily life, irrespective of meditative practice (Brown et al., 2007; Kabat-Zinn, 1990). Mindfulness has been shown to be an effective mechanism to support human flourishing and promotes happiness (Campos et al., 2016). A growing body of scientific literature also points to a correlation between mindfulness practice and prosocial behaviour (Donald et al., 2019) and improved social interactions (Adair et al., 2018). Additionally, it fosters the orientation of attention towards the present as participants can observe how the mind wanders and then deliberately shift the attention back again to the intended focus. This cultivates a multidimensional mode of mind that enables conscious, intentional movement between the discrepancy-based “doing” mode — a goal-oriented, processing mode to reduce the existing gap between a current state or situation and a desired state or situation — and the “being” mode — a contemplative, heightened awareness mode focused on experience and accepting of what is (Williams, 2008). One of the proposed seven dimensions of this “being” mode of mind is living in the past and future versus living in the present moment — or mental time travelling versus present orientation (Williams, 2008; Williams & Penman, 2011). Existing research exploring this dimension suggests that mindfulness is associated with openness towards, and acceptance of, the actual moment (Bishop et al., 2004), and that mindfulness is connected with enjoyment of the present moment (Nezlek et al., 2016).

The first study to explore the manifestation of mindfulness in language was Collins et al. (2009) in the context of an 8-week, mindfulness-based substance abuse intervention. A panel of experts generated a psycholinguistic dictionary containing lexical markers associated with both the mindfulness experience and the mindfulness journey, in order to develop a clinically valid way to assess levels of dispositional mindfulness. To date, we found that the relationship between mindfulness and language use has only been explored in seven studies, which are related to recollections of personal traumatic events (Moore & Brody, 2009), substance addiction treatment (Collins et al., 2009), increased physical activity tracking with mindfulness as a moderator (Tarachiu, 2014), working memory (Banks et al., 2015), promotion of underdeveloped cognitive-emotional processes in individuals with unhealthy attachment relationships (Caldwell & Shaver, 2015), changes in dispositional mindfulness after an 8-week intervention (Kaplan et al., 2018), and features of worry models (Bortoleto, 2019). These initial explorations have yielded mixed results: some report a mindfulness-related increase in the use of positive emotion words such as “fun” and “grateful” (Liehr et al., 2010), whilst other studies do not (Caldwell & Shaver, 2015; Kaplan et al., 2018); some report a mindfulness-related increase in use of present-moment words, for example, “is” and “am” (Caldwell & Shaver, 2015; Moore & Brody, 2009), whilst some studies did not find this (Bortoleto, 2019); and some report a mindfulness-related increase in word use related to cognitive processes such as “think” and “know” (Caldwell & Shaver, 2015; Moore et al., 2009), whereas other studies reported no change (Banks et al., 2015; Liehr et al., 2010). Common limitations of existing studies are the small size of language samples (all below 100,000 words), small participant groups (between 46 and 314 participants), and the short duration of the studies (2 days to 8 weeks with follow-up measures).

The potential value of addressing the manifestations of mindfulness in language use opens new avenues for research on the behavioural impact of mindfulness as a more naturalistic way of understanding and assessing mindfulness, which may complement available self-reporting methods such as questionnaires. For example, there is some evidence that those who engage in mindfulness practices may have lower levels of egocentric responses, and better emotional appraisal and social perception (Golubickis et al., 2016, 2022; Papies et al., 2012; Tan et al., 2014). What is not known is whether and how an interest in mindfulness, as a first step to explore linguistic manifestations of mindfulness, impacts how an individual expresses themselves through language. If mindfulness is linked to prosocial behaviour, then these findings might contribute to models to examine whether associated mindfulness linguistic markers are increasing or decreasing over a population. This study offers a first step in understanding the relationship between mindfulness and linguistic markers by analysing a large dataset comprising almost two million tweets (3.81 million words) published by 20,173 individuals on the online microblogging platform Twitter, between 26 March 2007 and 16 February 2020. This study aimed to explore the relationship between an interest in mindfulness and linguistic markers contained in entries on Twitter. This approach, if successful, may be used in future research to complement commonly used self-report methods with quantifiable behavioural metrics. The linguistic markers of interest in the current study were those associated with measures frequently used in mindfulness-related research: (1) dispositional mindfulness, (2) affective processes, (3) social orientation, and (4) mental time travelling versus present moment orientation as one dimension of the “being” mode of mind.

The linguistic markers of a group of Twitter users who follow public mindfulness figures (mindfulness interest group) were compared with a random selection of Twitter users (control group). This study tested four hypotheses: (1) that the mindfulness interest group uses language markers associated with mindfulness in their tweets more frequently than the control group; (2) that the tweets from the mindfulness interest group use a higher frequency of words associated with affective processes (including the subcategories of happiness and positive emotions) and a lower frequency of words associated with negative emotions than the control group; (3) that the mindfulness interest group more frequently uses language markers associated with social orientation including the subcategories of family and friend orientation in comparison to the control group; and (4) that, with regard to “time travelling versus present moment orientation”, an interest in mindfulness is associated with a higher frequency of language markers associated the “being” mode of mind (including the subcategory of present focus and a lower frequency of language markers associated with past focus, and future focus) in comparison to the control group.

## Method

### Twitter Sample

This study used a dataset of public Twitter messages posted by two groups: a mindfulness interest group (*n* = 10,347) and a control group consisting of a random selection of Twitter users from the Sentiment140 dataset (*n* = 9385). The aim of this selection process was to create participant categories of those who follow a public mindfulness expert, vs. a random sample of Twitter users. Twitter users were included in the mindfulness interest group if they followed at least one of five public mindfulness and meditation experts. The number of followers was collected at the time of selecting the mindfulness public figures for the study, so it may vary and only reflects a moment in time. The inclusion criteria to be considered publicly recognised mindfulness experts were (1) they had published books on mindfulness, (2) had a PhD, (3) were actively teaching on the subject of mindfulness or meditation at the time of the study, (4) were active on Twitter, and (5) had at least 30,000 followers at the time of selection (June 2019). The public mindfulness figures were identified by first creating a list of teachers from internationally renowned teaching and retreat centres including the Insight Meditation Society and Spirit Rock in North America or Gaia House in Europe, as well as from looking up the queries “top mindfulness teachers globally”, “global renowned mindfulness teachers”, “global mindfulness experts”, “most respected mindfulness teachers globally” and “best mindfulness teachers globally” on Google Search and extracting names from the resulting pages. This list was curated by looking up whether the teachers were published authors through a search on Amazon’s book section and Google Search using queries like the name and “ + book”. To identify if they held a PhD, Google Search was queried manually with search criteria like the name and “ + PhD” as well as the Wikipedia pages of the public figures. For those that met the “teaching”, “published author”, and “holds a PhD” criteria — and were alive — it was manually confirmed on Twitter whether they had an active account and how many followers they had. This number was finally used to stack rank them and identify the five that met these criteria and had the greatest number of followers. The mindfulness experts that met these criteria were Jack Kornfield (@JackKornfield, 131 k followers), Danny Penman (@DrDannyPenman, 110 k followers), Tara Brach (@tarabrach, 63.9 k followers), Jon Kabat-Zinn (@jonkabatzinn, 54.5 k followers), and Joan Halifax (@jhalifax, 36.2 k followers). The control group dataset was comparable in size to the mindfulness interest group. It included a random selection of users from the Sentiment140, a publicly available dataset that was accessed through the online data science platform Kaggle. Users with fewer than 20 public tweets were removed from the datasets for both groups.

Ethical approval was obtained from the Ethics Board at Bangor University prior to data collection. Public Twitter updates can be seen by anyone who has chosen to follow the publisher if they are not from password-protected conversations in moderated or closed groups. This means that it is reasonable to expect that Twitter users whose profile is public are likely to have no expectation of privacy. For this reason, neither the public figures nor the members of the mindfulness interest or control group were contacted directly for the study. This approach is in accordance with the British Psychological Society Ethics Guidelines for Internet-mediated Research (2017). In accordance with the Twitter Terms of Service and Privacy Policy (Twitter, 2021a, b, c), which users accept when creating an account, no demographic information was available about participants. These policies regulate the relationship between Twitter as a service provider and its users and clearly state that such information will not be made public. In addition, the data collection for this study complied with the Developer Terms (Twitter, 2021a, b, c). These terms define how Twitter content may be used, and specifically allow the analysis of aggregate data that does not store personal information such as user IDs.

### Procedure

All data were collected using the standard Twitter application programming interfaces or APIs (services to access and request Twitter data systematically). Specifically, the “GET followers/ids” endpoint was used to retrieve followers of the public mindfulness figures; the “GET users/lookup” endpoint was used to gather Twitter user information such as total number of followers or account creation date; and the “GET statuses/user_timeline” endpoint was used to obtain the content of the 200 most recent tweets (Twitter, 2021a, b, 1c). The Twitter APIs were accessed using the open-source programming language Python v3, and the wrapper Twython for Python (McGrath, 2014), which is a set of functions used for programming convenience.

The follower list for each of these five public figures was obtained in batches of 75,000 every 15 min. Once the complete list had been collected, the list of followers was randomised. A maximum of 200 of the most recent, public tweets were downloaded for each user from a random sample of 15,000 followers, at a rate of 900 API calls every 15 min. The dataset for the control group was accessed through Kaggle using the search query “Twitter” and activating the filter “dataset”. The resulting dataset, Sentiment140 (Go et al., 2009), was built by sending recurring search queries to the Twitter API for emoticons:) and:( and it is formed of the individual tweet results that the service returned. The downloadable csv file contains a total of 1.6 million tweets along with their labels and metadata that includes the user who published the tweet. A sample of 15,000 of these Twitter users was randomised and a maximum of the 200 most recent, public tweets per user was downloaded, at a rate of 900 API calls every 15 min.

Additionally, prior to analysis, software was used to clean the text in both groups and discard retweets (publication of content coming from other Twitter users), responses, tweets in languages other than English, and media files (images or videos). The content of the tweets was analysed to replace URLs with the text “[URL]”, and mentions of Twitter users with the text “[USER]”, as a means of preserving privacy. In order to avoid bias, no attempt was made manually or by computer programme to post-process the control group on the basis of having tweeted mindfulness-related content or following any or all of the five public figures in question.

### Measures

Linguistic Inquiry and Word Count (LIWC) built-in dictionaries were used to measure and compare the frequency of linguistic markers associated with affective processes, social orientation, and the “being” mode of mind. These dictionaries consist of words, word stems, emojis, and verbal constructions that have been iteratively identified and tested by experts to reflect psychological categories (Pennebaker et al., 2015). To measure linguistic markers of affective processes, this study examined the frequency of words in the LIWC “affective processes” category including the subcategories of “positive emotions” and “negative emotions”. It operationalised a metric for “happiness” by calculating the difference in the frequency of use of words in the positive and negative emotion subcategories (Happiness = positive emotions − negative emotions) (see example words in Table 1). Social orientation was measured computing the frequency of words in the LIWC “social processes” category. Within this category, the subcategories of “friend orientation” and “family orientation” were explored to provide granularity on social orientation (see Table 1 for example words). To explore linguistic markers associated with the “being” mode of mind, the study examined the frequency of words in the LIWC “Mental time travelling versus present moment orientation” category. Specifically, the subcategories of “time”, “present focus”, “past focus”, and “future focus” were explored.

**Table 1:** Example words in the categories and subcategories explored in this study

Linguistic categories	Example words
1. Mindfulness	curiosity, open, accepting
2. Affective processes	happy, sadly, yay
2.1. Happiness	nice, nasty
2.2. Positive emotions	nice, sweet, :)
2.3. Negative emotions	hurt, ugly, :(
3. Social orientation	interact, talk, everyone
3.1. Family orientation	daughter, dad, aunt
3.2. Friend orientation	buddy, neighbour
4. “Being” mode of mind	does, had, will
4.1. Present focus	I’m, now, use
4.2. Past focus	walked, were, had
4.3. Future focus	will, going to
5. Time	since, wait, clock

In addition, a third-party dictionary developed by a panel of experts (Collins et al., 2009) which includes words describing the mindfulness experience (e.g., “observe”, “accept”, “calm”) as well as challenges associated with the practice (e.g., “react”, “autopilot”, “judge”) was used to measure and compare the frequency of linguistic markers associated with dispositional mindfulness among Twitter users in the dataset. Table 1 shows examples of words in this category.

### Data Analyses

Linguistic analyses were performed with the 2015 version of the software LIWC, which is available in open access (Pennebaker et al., 2015). Psycholinguistics researchers developed this programme to analyse human, social, and psychological states through language. LIWC calculates the use frequency of certain specific words, word stems, or emoticons that are associated with psychological and cognitive processes, and it compares them against psychometrically validated categories, transforming text into numerical values. These values are expressed as the percentage of the total number of words in a text that belong to a specific category or subcategory, whilst controlling for absolute text length. For example, LIWC includes the subcategory “sadness” nested under “negative emotions” and, in turn, under the category “affective processes”. An output value of 56.15 for the category “sadness” means that 56.15% of the words in the analysed text are included in the “sadness” category. The language markers in the “sadness” category are also included, without limitation, in the parent category “negative emotions”, and in the overarching category “affective processes”. In addition to the built-in categories, LIWC 2015 can also be used to process text using custom-made dictionaries built by users, to explore other psychometric categories or for data in languages other than English.

Prior to conducting statistical analyses, all variables were reviewed to confirm a normal distribution. Then, descriptive statistics were calculated and independent *t*-tests were conducted to analyse the differences between the mindfulness interest group and the control group in relation to the variables of interest. For rigour, twelve separate linear regression analyses (weighted least squares) were performed to determine whether belonging to the mindfulness interest group had an effect on the variables explored when controlling, for each participant, the following eight factors as dependent variables: the date of their first and last tweets in the dataset, the number of replies, retweets, followers, and accounts followed, the word count of tweets, and when the Twitter account was first created. Lastly, when *t*-test results were statistically significant, the standardised mean difference (Cohen’s *d*) was estimated to assess effect size. Given the large sample, a significance criterion of *p* < 0.001 was adopted for all analyses.

## Results

Hypothesis 1 was that the mindfulness interest group would use language markers associated with mindfulness in their tweets more frequently than the control group. Consistent with the hypothesis, the use of lexical markers associated with mindfulness (full list of markers can be found in Collins et al., 2009) was significantly higher in the mindfulness interest group than in the control group (mean difference = 0.51; *p* < 0.001; Cohen’s *d* = 0.38). This difference was also statistically significant (*p* < 0.001) when other factors were controlled for (Tables 2 and 3), with a small positive effect size.

**Table 2:** Results of the independent *t*-test analysis examining differences between the mindfulness interest group and control group for the linguistic markers associated with dispositional mindfulness, affective processes, social orientation, and “being” mode of mind

Linguistic categories	Mindfulness interest group		Control group		*t*	*p*	Cohen’s *d*
	*M*	*SD*	*M*	*SD*			
Mindfulness	2.31	1.62	1.81	0.77	27.53	< 0.001*	0.38
Affective processes	6.83	2.34	6.74	2.34	2.77	0.006	0.04
Happiness	3.76	2.71	2.96	2.26	22.30	< 0.001*	0.32
Positive emotions	5.26	2.30	4.83	2.00	14.11	< 0.001*	0.20
Negative emotions	1.50	1.05	1.86	1.14	− 23.23	< 0.001*	− 0.33
Social orientation	11.25	3.57	10.00	3.38	25.09	< 0.001*	0.35
Family orientation	0.32	0.47	0.42	0.51	− 13.65	< 0.001*	− 0.19
Friend orientation	0.29	0.37	0.40	0.54	− 15.68	< 0.001*	− 0.22
Past focus	1.80	1.11	2.59	1.20	− 48.17	< 0.001*	− 0.65
Present focus	9.10	2.77	9.80	2.72	− 17.78	< 0.001*	− 0.25
Future focus	1.01	0.60	1.28	0.72	− 28.92	< 0.001*	− 0.40
Time	5.02	1.98	6.02	2.07	− 34.52	< 0.001*	− 0.48

*LIWC*, Linguistic Inquiry and Word Count; *M*, mean; *SD*, standard deviation

^*^Statistical significance *p* < 0.001

**Table 3:** Results of the linear regression analysis of LIWC variables with regard to group belonging

LIWC category	*β*	*p*	*SE*	*R* square
Mindfulness	0.11	< 0.001*	0.03	0.08
Affective processes	0.10	0.011	0.04	0.08
Happiness	0.67	< 0.001*	0.05	0.05
Positive emotions	0.38	< 0.001*	0.04	0.07
Negative emotions	− 0.29	< 0.001*	0.02	0.07
Social orientation	0.89	< 0.001*	0.06	0.09
Family orientation	− 0.08	< 0.001*	0.01	0.02
Friend orientation	− 0.08	< 0.001*	0.01	0.02
Past focus	− 0.64	< 0.001*	0.02	0.15
Present focus	− 0.68	< 0.001*	0.05	0.09
Future focus	− 0.18	< 0.001*	0.01	0.10
Time	− 0.82	< 0.001*	0.04	0.07

*β*, standard coefficient; *SE*, standard error

^*^Statistical significance

Hypothesis 2 was that the mindfulness interest group would use a higher frequency of words associated with affective processes (including the subcategories of happiness and positive emotions) and a lower frequency of words associated with negative emotions than the control group. Contrary to the hypothesis, the difference in use of linguistic markers associated with affective processes in tweets between the mindfulness interest group and the control group was not statistically significant (mean difference = 0.09; *p* = 0.006; Cohen’s *d* = 0.04). However, linear regression analysis revealed that belonging to the mindfulness interest group did not have a statistically significant effect on the variable affective processes (*p* = 0.011) when controlled for by other factors (Tables 2 and 3).

However, results for the three subcategories within affective processes were consistent with the hypothesis that tweets from the mindfulness interest group would present a higher frequency of linguistic markers associated with happiness (mean difference = 0.80; *p* < 0.001; Cohen’s *d* = 0.32), positive emotions (mean difference = 0.43; *p* < 0.001; Cohen’s *d* = 0.20), and a lower frequency of linguistic markers associated with negative emotions (mean difference =  − 0.36; *p* < 0.001; Cohen’s *d* =  − 0.33), when compared with the control group. Results for all three dimensions were statistically significant, with small effect sizes. Effect sizes were positive for happiness and positive emotions, and negative for negative emotions. Linear regression analysis indicated that belonging to the mindfulness interest group had a statistically significant effect (*p* < 0.001) on the three dimensions of interest when controlling for other factors (Tables 2 and 3).

Hypothesis 3 was that the mindfulness interest group would more frequently use language markers associated with social orientation including the subcategories of family and friend orientation in comparison to the control group. The results supported Hypothesis 3 (mean difference = 1.24; *p* < 0.001; Cohen’s *d* = 0.35). The results for this overarching category were statistically significant with a small effect size. Linear regression analysis revealed that this effect was also statistically significant when other factors were controlled for (*p* < 0.001).

However, when looking at the two subcategories of interest within the social orientation category, the results were contrary to the hypothesis. The mindfulness interest group presented a significantly lower frequency of linguistic markers associated with family orientation (mean difference =  − 0.10; *p* < 0.001; Cohen’s *d* =  − 0.19) and friend orientation (mean difference =  − 0.10; *p* < 0.001; Cohen’s *d* =  − 0.22) than the control group. Linear regression analysis indicated that belonging to the mindfulness interest group had a statistically significant effect (*p* < 0.001) for the variables in this category, when controlled against other factors (Tables 2 and 3).

Lastly, Hypothesis 4 was that an interest in mindfulness would be linked to a higher frequency of language markers associated with the “being” mode of mind (including the subcategory of present focus and a lower frequency of language markers associated with past focus, and future focus) in comparison to the control group. The “being” mode of mind was explored through one of its dimensions: mental time travelling versus present moment orientation (Williams & Penman, 2011). The LIWC variables “present focus”, “past focus”, “future focus”, and “time” were analysed. As hypothesised, the mindfulness interest group showed a significantly lower use of lexical markers associated with orientation towards the past (mean difference =  − 0.79; *p* < 0.001; Cohen’s *d* =  − 0.65) and the future (mean difference =  − 0.27; *p* < 0.001; Cohen’s *d* =  − 0.40) compared with the control group, with negative medium and small effect sizes, respectively.

However, contrary to the hypothesis, the mindfulness interest group showed a significantly lower use of language markers associated with present moment orientation (mean difference =  − 0.70; *p* < 0.001; Cohen’s *d* =  − 0.25) than the control group, with a small negative effect size. In addition, the data showed that the mindfulness interest group used language markers associated with time significantly less frequently than the control group (mean difference =  − 1.00; *p* < 0.001; Cohen’s *d* =  − 0.48), with a small negative effect size. Results for the four time-travelling variables (past focus, present focus, future focus, and time) were also statistically significant (*p* < 0.001) when controlled for other factors (Tables 2 and 3).

## Discussion

The present study examined the association between an interest in mindfulness and language markers used on the social media platform Twitter, specifically in relation to the categories of mindfulness, affective processes (happiness, positive emotions, negative emotions), social orientation (family orientation, friend orientation), and the “being” mode of mind (past focus, present focus, future focus, time). The linguistic analysis of 1.87 million microblogging entries (tweets) across 19,732 users was performed with the text analysis software LIWC.

The results in relation to the linguistic markers associated with mindfulness were as expected (Hypothesis 1), people with an interest in mindfulness used language markers associated with mindfulness more frequently. Although a statistically significant difference between the two groups for the broad category of affective process was not found (Hypothesis 2), the three subcategories within the affective process category all showed a statistically significant difference between the two groups. The mindfulness interest group used words related to happiness and positive emotions more frequently, and used language markers associated with negative emotions less frequently than the control group consisting of a random selection of Twitter users, even when controlling for other factors. The greater use of linguistic markers associated with mindfulness and affective processes for those with an interest in mindfulness are consistent with, and add to, the existing body of literature that explores the behavioural expression of the connection between mindfulness and psychological well-being (Brown & Ryan, 2003; Campos et al., 2016; Keng et al., 2011).

There was partial support for Hypothesis 3 relating to linguistic markers of social orientation and the “being” mode of mind. Whilst the group with an interest in mindfulness did show a significantly higher use of linguistic markers associated with social orientation, when the subcategories of family and friend orientation were explored, the mindfulness interest group showed a significantly lower use of these language markers. With regard to the “being” mode of mind, as hypothesised, an interest in mindfulness was associated with a significantly lower use of linguistic markers associated with past and future focus. However, contrary to the hypothesis, the mindfulness interest group had lower use of linguistic markers associated with the present focus in comparison to the control group.

The finding relating to a greater use of language markers associated with social orientation for those with an interest in mindfulness is in line with existing research which has shown that trait and state mindfulness is associated with enhanced interpersonal communication and social interactions (Adair et al., 2018; Burgoon et al., 2000; Donald et al., 2019). The higher use of social orientation language markers combined with a lower frequency of linguistic markers from the friends and family subcategories could be related to the concept of nonduality in mindfulness (Kabat-Zinn, 1990). This concept posits the dissolution of the subject-object separation with an embodied experience of “wholeness” where everyone and everything is interdependent, contrary to an in-group out-group experience (Husgafvel, 2018). This could suggest that those with an interest in mindfulness would be less prone to othering (with the “family” and “friends” categories being in-group versus out-group) and would instead use more interconnectedness-oriented language (a wholeness, interdependent view of the world). This is currently speculative, and more research is needed to explore this.

With regard to Hypothesis 4, the finding that the mindfulness interest group showed significantly less frequent use of language markers associated with the past and future compared to the control group is consistent with the hypothesis and existing research exploring behavioural manifestations of mindfulness and time-related language. For example, Caldwell and Shaver (2015) found a reduction in the use of past tense words in narratives on stressful or traumatic childhood experiences after mindfulness training. In addition, Hafenbrack et al. (2014) found that mindfulness was associated with a decreased focus of thoughts on the past and future and this was associated with reductions in negative affect. It was surprising that, contrary to Hypothesis 4, the mindfulness interest group was significantly less likely to use lexical markers associated with the present and with time more generally, and this is in contrast to findings from previous research (Caldwell & Shaver, 2015; Moore & Brody, 2009). A possible reason may be that mindfulness brings an orientation towards the present, that is, towards each individual moment in time, rather than in relation to the past or future. As Jon Kabat-Zinn (2016) explained, mindful awareness is experienced in a different type of present that is “outside of time, in the eternal now” (p. 1239).

These findings expand the foundational scientific literature to explore linguistic patterns associated with mindfulness using source data from the social media platform Twitter. This type of data is increasingly relevant the more that people live their lives online may encourage further mindfulness-related research about how users express themselves naturally online. This potential relationship between mindfulness and the use of certain linguistic markers opens up the possibility of developing a new, more naturalistic, and quantifiable way of assessing mindfulness-related features to complement the self-reporting methods on which scientific research in the field tends to rely, and which can introduce an element of bias (Van Dam et al., 2018). Research using linguistic markers in the future could thus provide a more complete picture of how mindfulness may manifest behaviourally. The practical implications of the present study are relevant for social media companies in a time when online hate is on the rise, with potentially devastating consequences (Bilewicz & Soral, 2020). Given the apparent relationship between an interest in mindfulness with positive emotions and social orientation, this research could encourage social media companies to amplify a mindful discourse in their algorithms. However, substantial further research is needed.

### Limitations and Future Research

Perhaps the most salient limitation in this study is that it is unknown whether those who follow mindfulness public figures on Twitter are also likely to practise mindfulness, or to what extent. Additionally, it is unknown whether followers of public mindfulness figures do so because they are more broadly interested in positive mental health and well-being, rather than mindfulness per se. The results of this present study are therefore associated with an assumed interest in mindfulness based on participants following mindfulness public figures. In future research, it would be useful to include a measure of dispositional mindfulness or gather data on frequency of mindfulness practice. Additionally, a replication of this study but with different groups could help unpack whether it is solely followers of mindfulness public figures that have these linguistic markers. It is not clear from this study if the observed results are because of a common interest in mindfulness, or whether there could be an alternative explanation, such as an interest in wellness more generally. Future research could investigate different groups, for example, Twitter users that follow key “healthy living” public figures which are not associated explicitly with mindfulness to see if the effects observed in this study are specific to an interest in mindfulness.

As there is developing research interest in whether mindfulness practice increases pro-social behaviours (e.g., Golubickis et al., 2022), further exploration around the use of linguistic markers may be a useful additional methodological approach. First steps here might be for future research to establish whether there are correlations between the language markers explored in this study and established self-reporting measures of dispositional mindfulness or pro-social inclinations such as compassion for others (Brown & Ryan, 2003; López et al., 2018). The use of experimental study designs to explore whether there are changes in use of linguistic markers after attending a mindfulness-based programme or engaging in regular mindfulness practice may also further elucidate the links between language use and trait mindfulness.

The question of the general applicability of the results is a further limitation since the Twitter population is not demographically representative of the population as a whole. For example, Twitter users differ from the general population in that they tend to be younger, more educated, and politically liberal (Mellon & Prosser, 2017; Wojcik & Hughes, 2019). The demographic information that is available on Twitter is also limited, and precludes any analysis of the data by gender, age, geography, and so on. Lastly on demographics, a potential limitation may be the fact that public figures are only from European and North American countries and not globally representative, which may also include a bias in their follower base. It may be useful in future research to also compare the linguistic markers of followers of mindfulness public figures from different geographical locations, for example followers of mindfulness public figures based in Asia with those who follow those who are based in Europe and North America. Lastly, one of the criteria to select mindfulness experts is to hold a PhD which excludes other popular spiritually oriented mindfulness and meditation experts, or those teaching out of a particular religious or cultural context. This means the results may be specific to individuals interested in scientific or evidence-based framings of mindfulness and more research to verify the general applicability of results would be required.

A broader point to consider is how ideas about mindfulness are showing up in public discourse and understanding. Although this study did not look at this issue directly, from the perspective of linguistic analysis, whilst there is clarity about the sequential development of the LIWC built-in dictionaries, little is known about how Collins et al. (2009) developed the mindfulness dictionary used in this study and few studies have tested it since it was developed. It is therefore unknown how closely the mindfulness dictionary may relate to lay understanding about mindfulness, there is some evidence which suggests that there is little convergence on key facets of mindfulness (for example, acceptance) between lay understanding and the constructs used in common measures of mindfulness (Choi et al., 2021). For example, University students, when asked what mindfulness is, often associated mindfulness with controlling emotions, and rarely with psychological acceptance (Hitchcock et al., 2016). It must be noted as a further limitation that recent studies have shown inconsistencies in the relationship between self-reported emotion and LIWC metrics (Sun et al., 2020). More research focusing on the analysis of the lexical markers included in the different categories that are built into the LIWC software, and their suitability for mindfulness research, would be beneficial. In addition, the mindfulness dictionary would need to be rigorously evaluated from a psychometric perspective to confirm its reliability and validity. Furthermore, the development of additional, specific, mindfulness-related metrics such as the seven attitudinal foundations, such as acceptance and non-striving (Kabat-Zinn, 1990), all dimensions of the being mode of mind (Williams & Penman, 2011; Williams, 2008), and nonduality, would be a welcome addition to the research field.

A final limitation of the present study is the use of computerised linguistic word count analysis. Whilst this methodology reveals significant lexical patterns, it does not account for semantics, for complex linguistic features like sarcasm and situational context, or for more qualitative analyses. Future research could employ more sophisticated natural language processing techniques to get a deeper understanding not only of lexical features but also of semantics, context, etc. For example, programmatic content clustering could be used to understand the relationship between an interest in mindfulness and topics that Twitter users tweet about, or more advanced sentiment analysis tools could provide insights that account for tone, sarcasm, irony, and the like. This would contribute to the emergent discussions about popular conceptions of mindfulness (as contrasted to scholarly definitions), and to how ideas about mindfulness are showing up in public discourse. Applied research might explore the development of an absentminded writing detection system to alert individuals that they may want to take a pause before publishing or sending the text. Finally, the very limited existing research in this area is largely based on written language. By contrast, most mindfulness research is based on programmes that rely primarily on spoken language. Therefore, future research should be extended to spoken language to deepen understanding of how mindfully the general population speaks, or to detect and analyse any changes in speech before and after interventions. Such research could also extend our understanding of the characteristics of inquiry as a key element of mindfulness training (Crane et al., 2015).

## Data Availability

Data analysed as part of the mindfulness interest group are openly available at www.twitter.com. Data used in the control group are included in Go et al. (2009) and cited in the “References” section.
